# Scrotal Pain Alters Doppler Findings in Varicocele: A Prospective Evaluation

**DOI:** 10.3390/jcm15031013

**Published:** 2026-01-27

**Authors:** Halil Demirçakan, Ali Şahin, Hüseyin Gültekin, Kürşat Küçüker, Mesut Berkan Duran, Serdar Toksöz, Murat Gül

**Affiliations:** 1 Department of Urology, Çumra State Hospital, Konya 42500, Türkiye; drhalildemircakan@gmail.com; 2 Department of Urology, School of Medicine, Selcuk University, Konya 42130, Türkiye; drsahinacademics@gmail.com; 3 Department of Urology, Dr. Suat Günsel Girne University Hospital, Kyrenia 99000, Cyprus; huseyin_gultekin@windowslive.com; 4 Department of Urology, School of Medicine, Pamukkale University, Denizli 20070, Türkiye; kursat_kucuker@hotmail.com (K.K.); drberkanduran@gmail.com (M.B.D.); 5 Department of Urology, Sincan Education and Research Hospital, Ankara 06949, Türkiye; serdartoksoz@gmail.com; 6 Department of Andrology, School of Medicine, Selcuk University, Konya 42130, Türkiye

**Keywords:** varicocele, ultrasonography, scrotal pain

## Abstract

**Objectives:** This study aimed to investigate the impact of scrotal pain on venous diameter and reflux duration in varicocele, and to assess the predictive value of ultrasonographic findings for varicocele grading. **Methods:** Fifty-two symptomatic patients with left-sided varicocele, presenting with infertility or scrotal pain, were prospectively evaluated. Grading was based on physical examination. Visual Analog Scale (VAS) scores, venous diameters, and reflux durations were measured using scrotal color Doppler ultrasonography (CDUS) both during active pain and after pain had markedly subsided or resolved. **Results:** After pain resolution, venous diameters significantly decreased in both resting and Valsalva states (*p* < 0.001). In grade-specific analysis, this reduction was significant only in grade II varicocele (rest: *p* = 0.004; Valsalva: *p* = 0.026). Reflux durations also significantly decreased after pain relief in all varicocele grades, both at rest and during Valsalva (*p* < 0.001 for all, except G3 Valsalva: *p* = 0.001). Ultrasonographic parameters during the pain-present state showed better discrimination for detecting grade I varicocele (AUC: 0.88), while the pain-free state provided better diagnostic accuracy for grade III varicocele (AUC: 0.69). Combining measurements from both conditions further improved predictive accuracy, especially for grade III varicocele (AUC: 0.77). **Conclusions:** Scrotal pain significantly influences scrotal CDUS findings in patients with varicocele, leading to measurable differences in venous diameter and reflux duration between pain-present and pain-free states. Therefore, consideration of symptom status when interpreting scrotal CDUS results may improve diagnostic accuracy and support more informed clinical decision-making.

## 1. Introduction

Varicocele is defined as the abnormal dilatation of the pampiniform venous plexus in the scrotum. It is a pathological condition characterized by impaired testicular drainage due to venous reflux in the internal spermatic vein [1]. The diagnosis of varicocele is established through physical examination [2]. According to the Dubin and Amelar classification system, which is based on physical examination, varicocele is graded as grade I, grade II, or grade III [3]. The World Health Organization has added subclinical varicocele, which cannot be detected by physical examination or the Valsalva maneuver but can be identified via scrotal color Doppler ultrasonography (CDUS), to this classification [4]. The guidelines of the European Association of Urology and the American Urological Association do not recommend the routine use of scrotal CDUS for the diagnosis of varicocele. They suggest using scrotal CDUS only in clinically suspicious cases or when physical examination is inconclusive [5,6,7,8]. Measurements of dilated vein diameters and reflux duration using scrotal CDUS can be performed in the standing or supine position, either at rest or during the Valsalva maneuver [9]. Venous dilatation and venous reflux disrupt the scrotal heat-exchange mechanism, leading to an increase in intratesticular temperature and subsequently impairing testicular function, which, over time, results in deterioration of semen parameters. Therefore, accurate ultrasonographic assessment of these parameters is considered critical for predicting the impact of varicocele on fertility [10].

Scrotal pain is observed in 2% to 10% of patients with varicocele [11]. The pathogenesis of scrotal pain in varicocele involves the compression of large neural fibers by dilated venous complexes, increased scrotal temperature, oxidative stress, and tissue ischemia secondary to venous stasis [12]. Once scrotal pain is confirmed to be caused by varicocele, conservative treatments should be implemented, including limitation of physical activities, scrotal elevation, and the use of nonsteroidal anti-inflammatory drugs (NSAIDs) [13,14]. Several clinical and technical factors influencing vein diameter and reflux duration measured by scrotal CDUS have been reported in the literature; these include performing measurements in the supine or standing position, acute exercise, and the time interval between ejaculation and the examination [15,16,17]. However, there are no studies examining the relationship between scrotal pain status and ultrasonographic findings in varicocele patients. We hypothesize that scrotal pain, by causing contraction of testicular cremasteric fibers, may impair venous return and thereby increase the diameter of varicose veins and the degree of reflux [18].

In this study, we aimed to investigate the effect of the presence and absence of scrotal pain on varicose vein diameter and reflux duration, as well as the ability of ultrasonographic findings to distinguish between varicocele grades.

## 2. Materials and Methods

### 2.1. Study Population and Study Design

This study included 52 patients diagnosed with varicocele who presented to the Urology Outpatient Clinic of Sincan Training and Research Hospital with complaints of infertility or scrotal pain between 15 August 2024 and 30 September 2024. This study was prospectively designed. It adhered to the principles of the Declaration of Helsinki, and informed consent was obtained from all participants. The study was approved by the local ethics committee (Approval No: E.562591, 6 August 2024). The reporting of this study conforms to the Strengthening the Reporting of Observational Studies in Epidemiology (STROBE) guidelines [19].

All patients were evaluated at the time they presented to our center. Patients were categorized into three groups based on the grade of varicocele determined during physical examination performed by a single experienced urologist at the time of presentation (Grade I, Grade II, and Grade III). Patients were not further classified according to the affected venous district (varicocele subtype), as this was not systematically assessed or recorded in the study protocol.

Only patients who reported recurrent, intermittent episodes of scrotal pain rather than a single isolated pain episode were included in the study. The “symptomatic state” was defined as the presence of scrotal pain reported by the patient at the time of presentation and confirmed during clinical evaluation. The pain-free state was defined as the time when the patient reported complete resolution of scrotal pain and was clinically asymptomatic. No formal minimum visual analog scale (VAS) threshold was required; however, pain had to be clinically relevant enough to prompt medical consultation. The interval between the symptomatic and pain-free scrotal CDUS assessments was planned to be approximately one week, and in practice, this interval showed small individual variations among patients. External factors that may influence venous caliber, such as physical activity, prolonged standing, and timing of sexual activity, could not be systematically standardized. All patients experienced both symptomatic and pain-free periods and completed scrotal CDUS assessments in both states; therefore, all participants were included in the within-subject analysis.

Patients with a history of inguinal or scrotal surgery, those with pathologies other than varicocele that could cause scrotal pain, those not reporting pain, or those who did not comply with the prescribed analgesic treatment were excluded from the study.

### 2.2. Ultrasonographic Evaluation

At the time of the initial evaluation—when patients reported active scrotal pain, and this was confirmed during clinical interview and physical examination—all individuals underwent pain assessment using the Visual Analog Scale (VAS) and scrotal CDUS. Scrotal CDUS was performed in all patients using the same ultrasound system (Mindray DC-80A) with a high-frequency 10 MHz linear transducer, and spectral Doppler settings were standardized across examinations with a wall filter of 50 Hz. All examinations were performed in the upright position using a subinguinal approach, and all measurements were obtained both at rest and during the Valsalva maneuver.

In all cases, venous diameter (mm) and reflux duration (s) were measured based on the largest dilated vein within the pampiniform plexus. The Valsalva maneuver was standardized by instructing patients to take a deep inspiration followed by forced expiration against a closed glottis with contraction of the abdominal muscles for approximately 5 s.

For the diagnosis of varicocele, a reflux duration > 2 s was accepted as the threshold, and a venous diameter ≥ 3 mm was considered diagnostic. These thresholds were defined in accordance with the recommendations of the European Society of Urogenital Radiology–Scrotal and Penile Imaging Working Group (ESUR-SPIWG) [7].

Bilateral scrotal examinations were performed in all patients, and both testes were evaluated as part of routine clinical assessment; however, contralateral findings and testicular volume measurements were not systematically recorded as study variables and were therefore not included in the statistical analysis.

All patients were recalled for re-evaluation after one week of standardized oral NSAID treatment. Once patients reported resolution of scrotal pain, they were re-evaluated in the pain-free state using the same ultrasonographic protocol. VAS scores, venous diameters, and reflux durations were measured again under identical conditions. All scrotal CDUS examinations were performed by a single experienced uroradiologist working during routine office hours. Ultrasonographic and clinical parameters recorded during the pain-present and pain-free periods were statistically compared.

### 2.3. Statistics

Frequency and percentage values were calculated for categorical variables, and differences between categorical features were analyzed using the chi-square test. For numerical variables, the mean, standard deviation, and confidence intervals were determined. The Kolmogorov–Smirnov test was employed to assess data distribution. For normally distributed binary data, Student’s t-test and paired-samples t-test were utilized, while the Mann–Whitney U-test was applied to analyze non-normally distributed data. For non-binary numerical variables, ANOVA was used for normally distributed data, while the Kruskal–Wallis test was employed for non-normally distributed data. Post hoc analyses were performed using the Bonferroni post hoc test. A *p*-value of less than 0.05 was considered statistically significant. All statistical analyses, except for logistic regression, were conducted using the DATAtab online software (Graz, Austria: DATAtab, 2025; https://www.datatab.net/ (accessed on 17 August 2025)). Logistic regression, ROC curve generation, and AUC calculations were performed using the Python programming language (version 3.10.2) with the libraries ‘pandas’ (version 1.5.3), ‘scikit-learn’ (version 1.1.3), and ‘matplotlib’ (version 3.6.3).

## 3. Results

### 3.1. Patient Characteristics

A total of 52 symptomatic left-sided varicocele patients were prospectively included in the study. The mean age of participants was 26.2 ± 5.4 years, 95% CI [24.71–27.72], with no significant differences in age among the varicocele grades (*p* = 0.072). Based on physical examination, 13 patients (25%) were classified as grade I, 29 (55.77%) were classified as grade II, and 10 (19.23%) were classified as grade III. Additionally, all varicoceles in the study cohort were localized to the left pampiniform plexus. The mean interval between the pain-present and pain-free scrotal CDUS assessments was 7.85 ± 1.13 days and did not differ significantly across varicocele grades (*p* = 0.211) (Table 1).

At the time of initial assessment, when patients were experiencing scrotal pain, the mean Visual Analog Scale (VAS) score was 5.33 ± 1.23 (95% CI: 4.98–5.67). No significant differences were observed in VAS scores among varicocele grades during this pain-present period (*p* = 0.847). Following a period during which patients reported absence of scrotal pain, the overall mean VAS score significantly decreased to 1.6 ± 1.36 (95% CI: 1.22–1.98; *p* < 0.001). Similarly, in the pain-free state, no significant differences were found between varicocele grades regarding VAS scores (*p* = 0.715). Paired comparisons confirmed that the reduction in VAS scores from the painful to pain-free period was statistically significant across all varicocele grades (Supplementary Figure S1).

### 3.2. Ultrasonographic Measurements

In the pain-present state, venous diameters measured at rest significantly differed among all varicocele grades (*p* < 0.001; G1–2: *p* < 0.001, G1–3: *p* < 0.001, G2–3: *p* < 0.001). Similarly, venous diameters measured during the Valsalva maneuver while patients were experiencing pain also exhibited significant differences among the grades (*p* < 0.001).

In the pain-free state, venous diameters measured at rest continued to show significant differences across all grades (*p* < 0.001; G1–2: *p* = 0.047, G1–3: *p* < 0.001, G2–3: *p* = 0.003). Likewise, venous diameters during the Valsalva maneuver in the pain-free period also demonstrated significant differences among the grades (*p* < 0.001; G1–2: *p* = 0.03, G1–3: *p* < 0.001, G2–3: *p* = 0.01) (Table 1, Supplementary Figures S2–S5).

**Figure 1:**
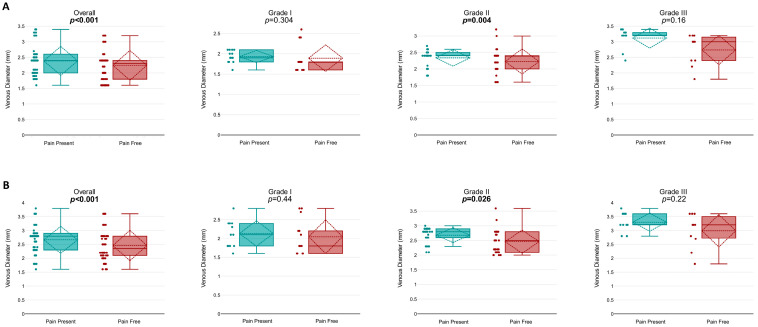
Comparison of venous diameters (VDs) for pain-present and pain-free states in different conditions and grades. (**A**) Resting venous diameters for pain-present and pain-free states across all grades (Overall and Grades I, II, and III). The overall analysis demonstrates a significant difference (*p* < 0.001). Subgroup analyses reveal significant differences for Grade II (*p* = 0.004), while Grades I and III show no statistical significance (*p* = 0.304 and *p* = 0.16, respectively). (**B**) Venous diameters during the Valsalva maneuver for pain-present and pain-free states across all grades. The overall analysis again demonstrates a significant difference (*p* < 0.001). Subgroup analyses show a significant change in Grade II (*p* = 0.026), with no significant differences in Grades I and III (*p* = 0.44 and *p* = 0.22, respectively). The boxplots illustrate the median, interquartile range (IQR), and outliers for each group.

When comparing venous diameters between the pain-present and pain-free states, a significant reduction in overall venous diameter was observed at rest following the resolution of pain (2.39 ± 0.47 mm vs. 2.24 ± 0.48 mm; *p* < 0.001). In grade-specific analysis, this reduction was statistically significant only in grade II varicocele patients (2.34 ± 0.26 mm vs. 2.23 ± 0.39 mm; *p* = 0.004), whereas no significant change was observed in grades I or III (*p* = 0.304 and *p* = 0.16, respectively) (Figure 1A).

During the Valsalva maneuver, a significant reduction in overall venous diameter was also observed after pain resolution (2.66 ± 0.49 mm vs. 2.46 ± 0.55 mm; *p* < 0.001). Grade-specific analysis again revealed a significant reduction only in grade II patients (2.68 ± 0.26 mm vs. 2.46 ± 0.4 mm; *p* = 0.026), while no significant differences were seen in grades I or III (*p* = 0.44 and *p* = 0.22, respectively) (Figure 1B).

In the pain-present state, the mean reflux duration at rest was 1.56 ± 0.85 s, with no significant differences among the grades (*p* = 0.057). In the pain-free state, the mean reflux duration at rest decreased to 0.46 ± 0.58 s, with significant differences between all grades except between grades I and II (*p* < 0.001; G1–2: *p* = 0.173, G1–3: *p* < 0.001, G2–3: *p* = 0.002). (Supplementary Figure S6). During the Valsalva maneuver, mean reflux duration was 2.37 ± 0.6 s in the pain-present state and 1.38 ± 0.57 s in the pain-free state. However, reflux durations during the Valsalva maneuver showed no significant differences among the grades in either condition (pain-present: *p* = 0.458; pain-free: *p* = 0.458).

**Figure 2:**
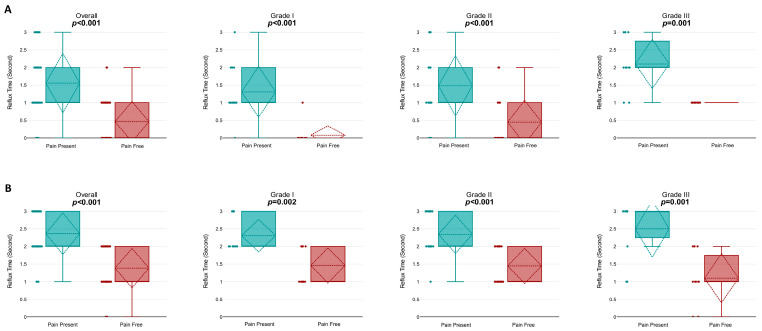
Comparison of reflux time (RT) in pain-present and pain-free states under different conditions and grades. (**A**) Resting reflux time for pain-present and pain-free states across all grades (Overall and Grades I, II, and III). The overall analysis indicates a significant reduction in reflux time (*p* < 0.001). Subgroup analyses demonstrate significant reductions for Grades I (*p* < 0.001), II (*p* < 0.001), and III (*p* = 0.001). (**B**) Reflux time during the Valsalva maneuver for pain-present and pain-free states across all grades. The overall analysis reveals a significant reduction (*p* < 0.001). Subgroup analyses show significant reductions for Grades I (*p* = 0.002), II (*p* < 0.001), and III (*p* = 0.001). Boxplots display the median, interquartile range (IQR), and outliers for each group.

Comparative analysis between pain-present and pain-free states revealed a significant reduction in overall reflux duration at rest (1.56 ± 0.85 s vs. 0.46 ± 0.58 s; *p* < 0.001). Grade-specific analysis indicated statistically significant reductions for all grades: grade I (*p* < 0.001), grade II (*p* < 0.001), and grade III (*p* = 0.001). (Figure 2A).

Similarly, during the Valsalva maneuver, a significant decrease in reflux duration was found after the resolution of pain (2.37 ± 0.6 s vs. 1.38 ± 0.57 s; *p* < 0.001). Again, significant reductions were detected for grade I (*p* = 0.002), grade II (*p* < 0.001), and grade III (*p* = 0.001). (Figure 2B).

### 3.3. Efficiency of Ultrasonographic Measurements in Predicting Varicocele Grades

To evaluate the predictive performance of ultrasonographic parameters for varicocele grading, logistic regression models were generated using data obtained during both pain-present and pain-free states. Receiver operating characteristic (ROC) curves were constructed, and the area under the curve (AUC) values were calculated for each grade.

When using ultrasonographic measurements recorded during the pain-present state, the AUC values for grade prediction were 0.88 for grade I, 0.59 for grade II, and 0.64 for grade III. These results suggest that ultrasonographic parameters taken while the patient is symptomatic may provide better discriminatory capacity for detecting lower-grade varicoceles.

In contrast, AUC values obtained from the pain-free state measurements were 0.77 for grade I, 0.54 for grade II, and 0.69 for grade III. This suggests an improvement in diagnostic accuracy for grade III varicocele when patients are evaluated in a symptom-free condition.

When measurements from both pain-present and pain-free states were combined in the logistic regression model, the AUC values were 0.83 for grade I, 0.59 for grade II, and 0.77 for grade III. These findings indicate that utilizing dual-time-point ultrasonographic data enhances the predictive accuracy for higher-grade varicoceles, particularly grade III.

**Figure 3:**
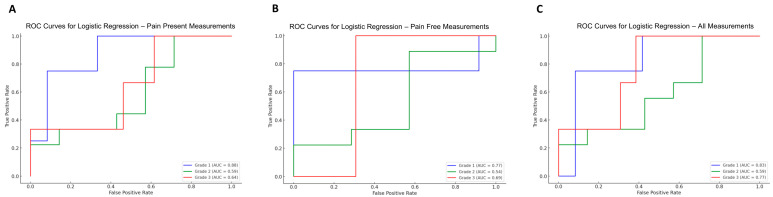
Receiver Operating Characteristic (ROC) curves for logistic regression models assessing classification performance across different grades. (**A**) ROC curves for pain-present measurements, displaying AUC values for Grade 1 (AUC = 0.88), Grade 2 (AUC = 0.59), and Grade 3 (AUC = 0.64). (**B**) ROC curves for pain-free measurements, displaying AUC values for Grade 1 (AUC = 0.77), Grade 2 (AUC = 0.54), and Grade 3 (AUC = 0.69). (**C**) ROC curves for all measurements combined, displaying AUC values for Grade 1 (AUC = 0.83), Grade 2 (AUC = 0.59), and Grade 3 (AUC = 0.77). The curves illustrate the trade-off between sensitivity (True Positive Rate) and specificity (1 − False Positive Rate) for each logistic regression model.

Overall, these results emphasize the dynamic nature of ultrasonographic findings in varicocele patients and support the use of both symptomatic and asymptomatic assessments to improve staging accuracy (Figure 3).

## 4. Discussion

This study focused on whether the presence or absence of scrotal pain influences ultrasonographic variables—namely vein diameter and reflux duration—across varying varicocele grades. To our knowledge, there are no other studies in the literature evaluating the relationship between ultrasonographic parameters and scrotal pain. According to our findings, both overall venous diameters and overall reflux durations measured during resting and Valsalva conditions significantly decreased during the pain-free state. In grade-specific analysis, venous diameters during resting and Valsalva conditions in the pain-free period significantly decreased only in patients with grade II varicocele, while reflux durations significantly decreased in all grades. However, because semen analysis and fertility outcomes were not evaluated in this study, it is not possible to draw conclusions regarding the prognostic impact of the ultrasonographic findings on reproductive function.

As a secondary analysis, we evaluated the performance of ultrasonographic parameters obtained during pain-present and pain-free periods in discriminating between varicocele grades. Our findings indicate that measurements obtained during the pain-present period provide higher accuracy in distinguishing grade I and grade II varicocele, whereas measurements obtained during the pain-free period are relatively more successful in distinguishing grade III varicocele. In addition, the combined evaluation of pain-present and pain-free measurements may offer additional benefit in discriminating grade III varicocele. The aim of these predictive models is not to redefine clinical staging, but rather to assess the potential for bias in physical examination–based clinical grading when ultrasonography-derived measurements are obtained at a single time point and are dependent on symptom status.

Various clinical and technical factors influencing vein diameter and reflux duration as measured by scrotal CDUS have been reported in the literature [15,16,17]. In a study by Kayra et al., venous diameter and reflux duration were measured in different projections (both testicular apex and subinguinal region) in supine and standing positions, and it was shown that venous diameter and reflux duration in the standing position were significantly higher compared to the supine position in both projections [15]. A study examining the effect of acute exercise on spermatic vein diameter and reflux duration demonstrated that overall venous diameter and reflux duration measured during the Valsalva maneuver significantly increased after the exercise test compared to baseline. In grade-specific analysis, venous diameter and reflux duration increased after the exercise test in patients with grade I or II varicocele, while no changes were observed in grade III varicocele [16]. Another study by Kızılkan et al. investigated the effect of the time interval between ejaculation and scrotal CDUS on varicose vein diameter and reflux duration, showing that varicose vein diameter and reflux grade could be higher when scrotal CDUS was performed within 90 min after ejaculation [17].

Scrotal pain may be associated with reflex contraction of the testicular cremasteric muscle fibers, a physiological response that has been described in association with venous congestion, larger venous diameter, and higher reflux measurements. Similarly, pain-free states may coincide with reduced cremasteric muscle tone and lower levels of venous congestion, together with lower venous diameter and reflux values [18]. These observations suggest that pain represents not only a symptom, but also a physiological state that is temporally associated with variations in the hemodynamic appearance of varicocele.

Conversely, pain may not only be a cause of increased venous dilatation and reflux, but also a consequence of these changes. Scrotal pain has been reported to be associated with mechanisms such as compression of perivenous nerve fibers by dilated venous complexes, increased venous pressure, local hypoxia, oxidative stress, elevated testicular temperature, hormonal imbalance, and reflux of adrenal or renal metabolites [14]. From this perspective, scrotal pain may be considered a clinical manifestation that can be triggered by factors that increase venous congestion, such as physical activity or prolonged standing. The coexistence of venous congestion and pain may reflect a dynamic interplay between these features, and symptomatic periods may be associated with higher venous diameter and reflux measurements, representing a potential source of methodological bias that should be considered when interpreting the results.

In addition to hemodynamic changes, inflammatory processes may also influence venous diameter and reflux duration; indeed, inflammatory mediators such as substance P, calcitonin gene–related peptide (CGRP), and cytokines have been reported to modulate venous tone and vascular permeability, thereby potentially affecting measured venous diameter and reflux parameters [20]. Because patients received NSAID treatment for pain palliation, changes in the inflammatory milieu may have coincided with symptom resolution and may have contributed to the observed ultrasonographic differences.

Additionally, the lack of systematic control or recording of time-varying external factors known to influence venous diameter and reflux, such as recent physical activity, prolonged standing, timing of ejaculation, and time of day, may have contributed to the observed within-subject differences and resulted in residual confounding that should be considered when interpreting the results.

From a clinical perspective, our findings indicate that scrotal CDUS parameters in patients with varicocele may vary according to symptom status and may differ between pain-present and pain-free periods. Ultrasonographic measurements obtained during periods of active scrotal pain may be transiently higher with respect to venous diameter and reflux duration, which may be associated with overestimation of disease severity, particularly in lower-grade varicocele. Conversely, assessments performed exclusively during pain-free periods may underestimate hemodynamic alterations that may be clinically relevant in symptomatic patients. We therefore believe that considering the presence of scrotal pain, particularly in patients with borderline or intermediate-grade varicocele, and repeating ultrasonographic evaluations at different time points may improve diagnostic assessment.

The clinical relevance of symptom-related ultrasonographic variability is primarily evident in adult practice, as scrotal CDUS findings are more frequently integrated into clinical decision-making in this population. In contrast, in adolescent and young adult patients, symptomatic varicocele is generally considered an indication for surgical intervention regardless of ultrasonographic thresholds [21]. Therefore, our findings are considered to be most informative for clinical practice in the adult patient population.

This study has several limitations. The number of patients included was relatively small, which may limit the generalizability of our findings. Moreover, baseline and follow-up semen analysis data and post-varicocelectomy pregnancy outcomes were unavailable, precluding assessment of the prognostic value of scrotal CDUS findings obtained during pain-present and pain-free periods with respect to fertility. Only symptomatic patients were included, and no asymptomatic comparison group was available. This limits our ability to determine whether the observed scrotal CDUS changes are specific to painful episodes or represent normal physiological variation in varicocele. Patients were not classified according to the affected venous district (varicocele subtype). Given that the varicocele subtype may influence clinical assessment and surgical outcomes, this limits our ability to determine whether the observed ultrasonographic variability is specific to particular varicocele subtypes and restricts the generalizability of our findings across different venous patterns.

Future prospective studies incorporating treatment outcomes and fertility parameters will help to clarify how symptom-related scrotal CDUS changes should be integrated into clinical algorithms.

## 5. Conclusions

This study highlights that scrotal pain can influence ultrasonographic findings invaricocele patients, with notable changes observed in venous diameter and reflux time between pain-present and pain-free states. These results suggest that symptom status should be considered when interpreting scrotal CDUS results. Evaluating patients in both symptomatic and asymptomatic conditions may enhance diagnostic accuracy and support better-informed clinical decisions. However, because semen analysis and reproductive outcomes were not assessed, these findings should not be interpreted in terms of fertility or reproductive prognosis.

## Figures and Tables

**Table 1:** Baseline demographic, clinical, and ultrasonographic parameters of patients with varicocele stratified by grade (Grade I, Grade II, and Grade III). Pain-present and pain-free assessments include Visual Analog Scale (VAS) scores for pain, venous diameters (VDs) at rest and during Valsalva, and reflux times at rest and during Valsalva. Significant *p*-values (<0.05) are indicated in bold. (^α^ ANOVA and ^β^ Kruskal–Wallis).

		Overall	Grade I	Grade II	Grade III	*p* Value
**Number, (%)**		52 (100)	13 (25)	29 (55.77)	10 (19.23)	-
Age		26.21 ± 5.4 [24.71–27.72]	25.85 ± 3.74 [23.59–28.11]	27.48 ± 6.03 [25.19–29.78]	23 ± 4.11 [20.06–25.94]	0.072 ^α^
Varicocele Location	Left	52 (100)	13 (100)	29 (100)	10 (100)	-
	Right	0 (0)	0 (0)	0 (0)	0 (0)	
USG timing (days) after the pain-presentation		7.85 ± 1.13 [7.53–8.16]	7.46 ± 1.05 [6.83–8.1]	7.86 ± 1.13 [7.43–8.29]	8.3 ± 1.16 [7.47–9.13]	0.211 ^α^
Pain-Present VAS		5.33 ± 1.23 [4.98–5.67]	5.31 ± 1.11 [4.64–5.98]	5.28 ± 1.22 [4.81–5.74]	5.5 ± 1.51 [4.42–6.58]	0.847 ^β^
Pain-Free VAS		1.6 ± 1.36 [1.22–1.98]	1.54 ± 0.78 [1.07–2.01]	1.72 ± 1.6 [1.12–2.33]	1.3 ± 1.25 [0.4–2.2]	0.715 ^β^
Pain-Present VD (Resting)		2.39 ± 0.47 [2.26–2.52]	1.93 ± 0.15 [1.84–2.02]	2.34 ± 0.26 [2.24–2.44]	3.12 ± 0.34 [2.88–3.36]	**<0.001 ^α^**
Pain-Free VD (Resting)		2.24 ± 0.48 [2.11–2.38]	1.89 ± 0.34 [1.69–2.1]	2.23 ± 0.39 [2.08–2.38]	2.74 ± 0.5 [2.38–3.1]	**<0.001 ^α^**
Pain-Present VD (Valsalva)		2.66 ± 0.49 [2.53–2.8]	2.13 ± 0.35 [1.92–2.34]	2.68 ± 0.26 [2.58–2.78]	3.3 ± 0.34 [3.05–3.55]	**<0.001 ^α^**
Pain-Free VD (Valsalva)		2.46 ± 0.55 [2.31–2.61]	2.05 ± 0.46 [1.77–2.33]	2.46 ± 0.4 [2.31–2.62]	2.99 ± 0.62 [2.55–3.43]	**<0.001 ^α^**
Pain-Present Reflux Time (Resting)		1.56 ± 0.85 [1.32–1.79]	1.31 ± 0.75 [0.85–1.76]	1.48 ± 0.87 [1.15–1.81]	2.1 ± 0.74 [1.57–2.63]	0.057 ^β^
Pain-Free Reflux Time (Resting)		0.46 ± 0.58 [0.3–0.62]	0.08 ± 0.28 [−0.09–0.24]	0.45 ± 0.63 [0.21–0.69]	1 ± 0 [1–1]	**<0.001 ^β^**
Pain-Present Reflux Time (Valsalva)		2.37 ± 0.6 [2.2–2.53]	2.31 ± 0.48 [2.02–2.6]	2.34 ± 0.55 [2.13–2.56]	2.5 ± 0.85 [1.89–3.11]	0.458 ^β^
Pain-Free Reflux Time (Valsalva)		1.38 ± 0.57 [1.23–1.54]	1.46 ± 0.52 [1.15–1.78]	1.45 ± 0.51 [1.26–1.64]	1.1 ± 0.74 [0.57–1.63]	0.345 ^β^

## Data Availability

The datasets used and/or analyzed during the current study are available from the corresponding author on reasonable request.

## Supplementary Materials

The following supporting information can be downloaded at https://www.mdpi.com/article/10.3390/jcm15031013/s1, Figure S1. Comparison of Visual Analog Scale (VAS) scores for pain-present and after pain-free VAS scores across all participants (Overall) and by varicocele grade (Grade I, Grade II, and Grade III). Box plots illustrate the median, interquartile range (IQR), and outliers for each group. Significant reductions in VAS scores were observed across all grades and the overall cohort (*p* < 0.001). The statistical significance was determined using paired-sample t-tests. Blue and red boxes represent pain-present and pain-free VAS scores, respectively; Figure S2. Comparison of pain-present venous diameters (VD) at rest across different varicocele grades. The boxplots represent the median, interquartile range (IQR), and outliers for Grades 1, 2, and 3. Statistical analysis shows significant differences in venous diameters between all grades (*p* < 0.001); Figure S3. Comparison of pain-present venous diameters (VD) during the Valsalva maneuver across different varicocele grades. The boxplots represent the median, interquartile range (IQR), and outliers for Grades 1, 2, and 3. Statistical analysis reveals significant differences in venous diameters between all grades (*p* < 0.001); Figure S4. Comparison of pain-free state venous diameters (VD) at rest across different varicocele grades. The boxplots depict the median, interquartile range (IQR), and outliers for Grades 1, 2, and 3. Statistical analysis indicates significant differences in venous diameters between Grades 1 and 2 (*p* = 0.047), Grades 2 and 3 (*p* = 0.003), and Grades 1 and 3 (*p* < 0.001); Figure S5. Comparison of pain-free venous diameters (VD) during the Valsalva maneuver across different varicocele grades. The boxplots illustrate the median, interquartile range (IQR), and outliers for Grades 1, 2, and 3. Statistical analysis reveals significant differences in venous diameters between Grades 1 and 2 (*p* = 0.03), Grades 2 and 3 (*p* = 0.01), and Grades 1 and 3 (*p* < 0.001); Figure S6. Comparison of pain-free reflux time (RT) at rest across different varicocele grades. The boxplots display the median, interquartile range (IQR), and outliers for Grades 1, 2, and 3. Statistical analysis shows significant differences in reflux time between Grades 1 and 3 (*p* < 0.001) and Grades 2 and 3 (*p* = 0.002), while the difference between Grades 1 and 2 is not significant (*p* = 0.173).
