# Supplementary material for: Scrotal Pain Alters Doppler Findings in Varicocele: A Prospective Evaluation
Source: J Clin Med. 2026 Jan 27;15(3):1013. doi: 10.3390/jcm15031013 (PMC12897755; doi:10.3390/jcm15031013)
Supplement: Supplementary file 1 [file jcm-15-01013-s001.zip › jcm-4099081-supplementary.pdf]

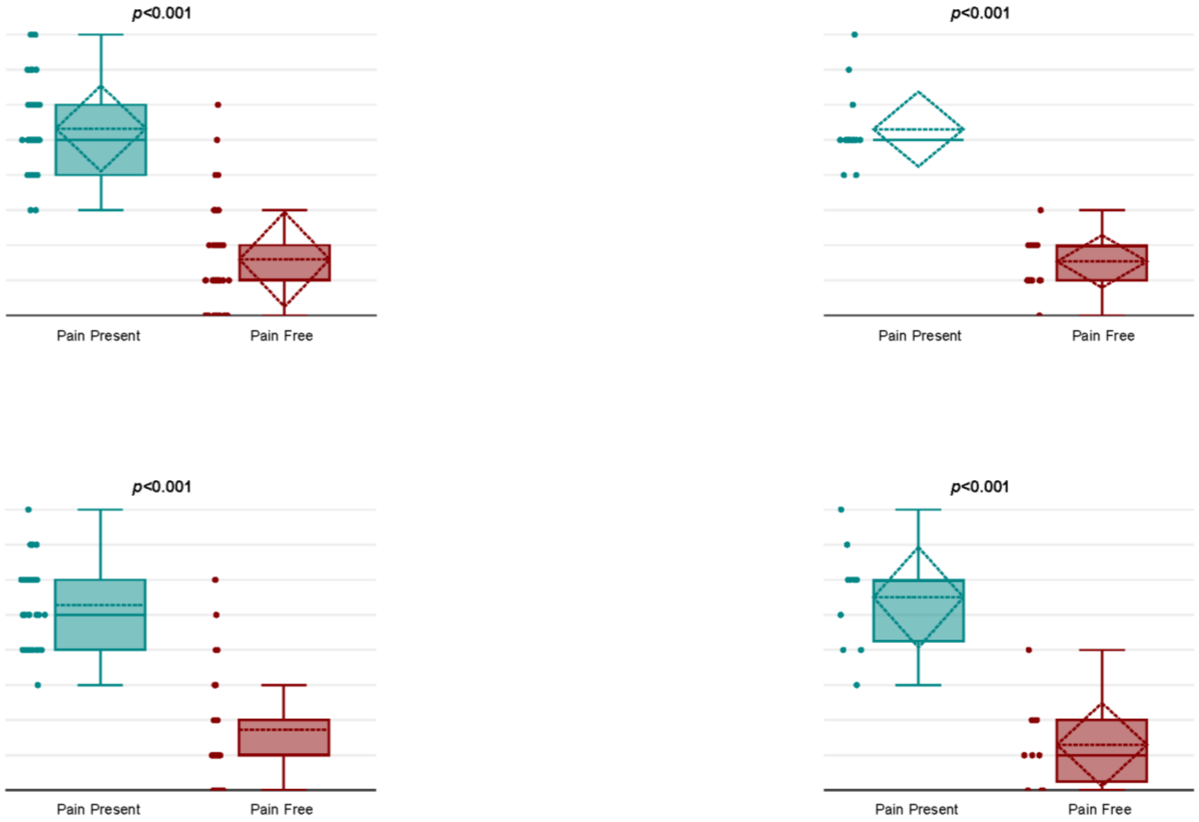

Supplementary Figure S1. Comparison of Visual Analog Scale (VAS) scores for pain-present and after pain-free VAS scores across all participants (Overall) and by varicocele grade (Grade I, Grade II, and Grade III). Box plots illustrate the median, interquartile range (IQR), and outliers for each group. Significant reductions in VAS scores were observed across all grades and the overall cohort ($p<0.001$). The statistical significance was determined using paired-sample t-tests. Blue and red boxes represent pain-present and pain-free VAS scores, respectively.

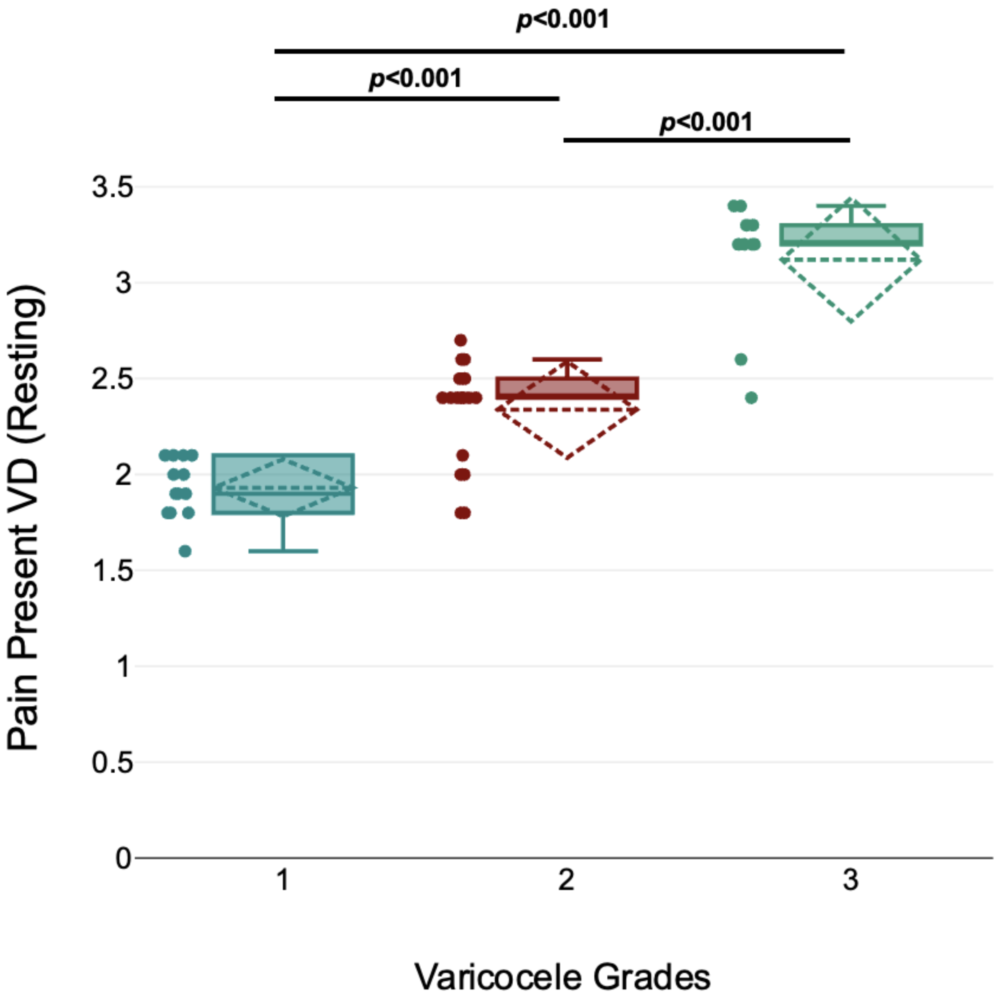

Supplementary Figure S2. Comparison of pain-present venous diameters (VD) at rest across different varicocele grades. The boxplots represent the median, interquartile range (IQR), and outliers for Grades 1, 2, and 3. Statistical analysis shows significant differences in venous diameters between all grades (p<0.001).

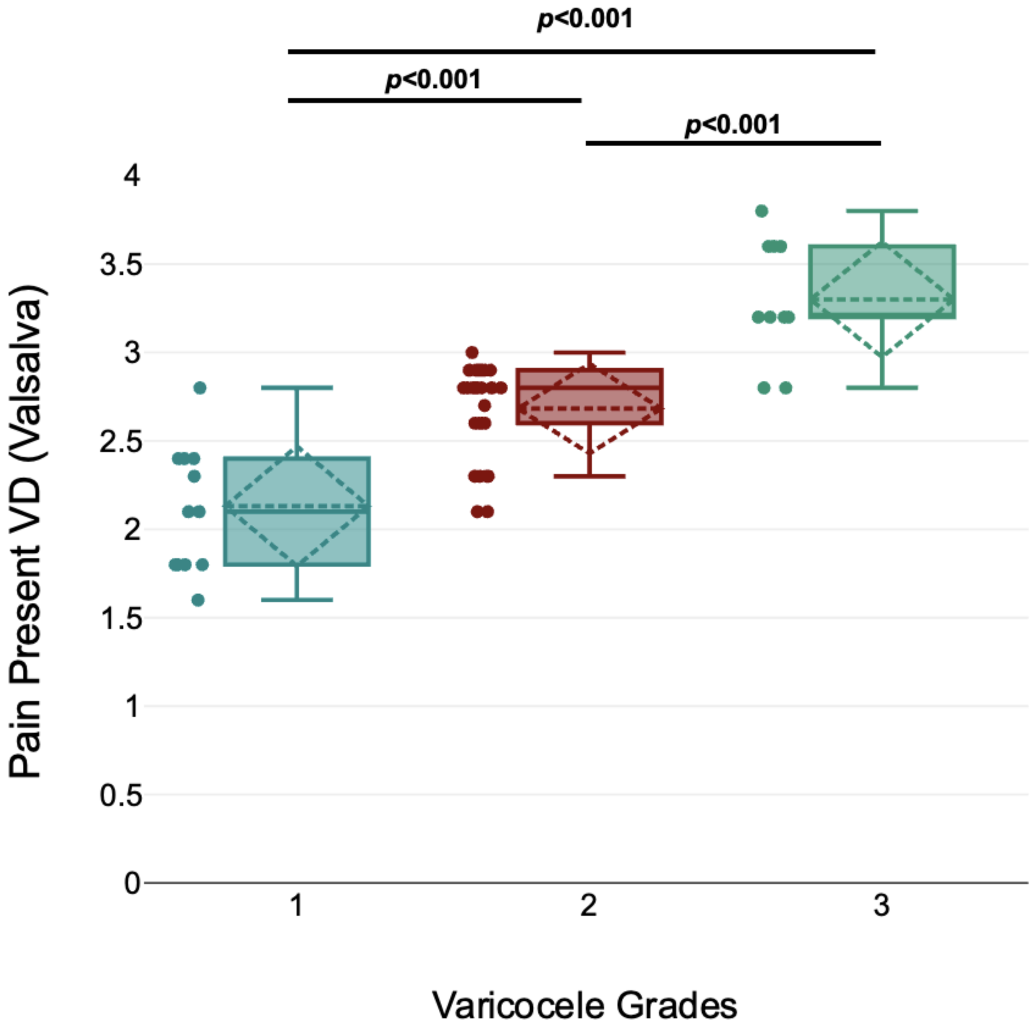

Supplementary Figure S3. Comparison of pain-present venous diameters (VD) during the Valsalva maneuver across different varicocele grades. The boxplots represent the median, interquartile range (IQR), and outliers for Grades 1, 2, and 3. Statistical analysis reveals significant differences in venous diameters between all grades (p<0.001).

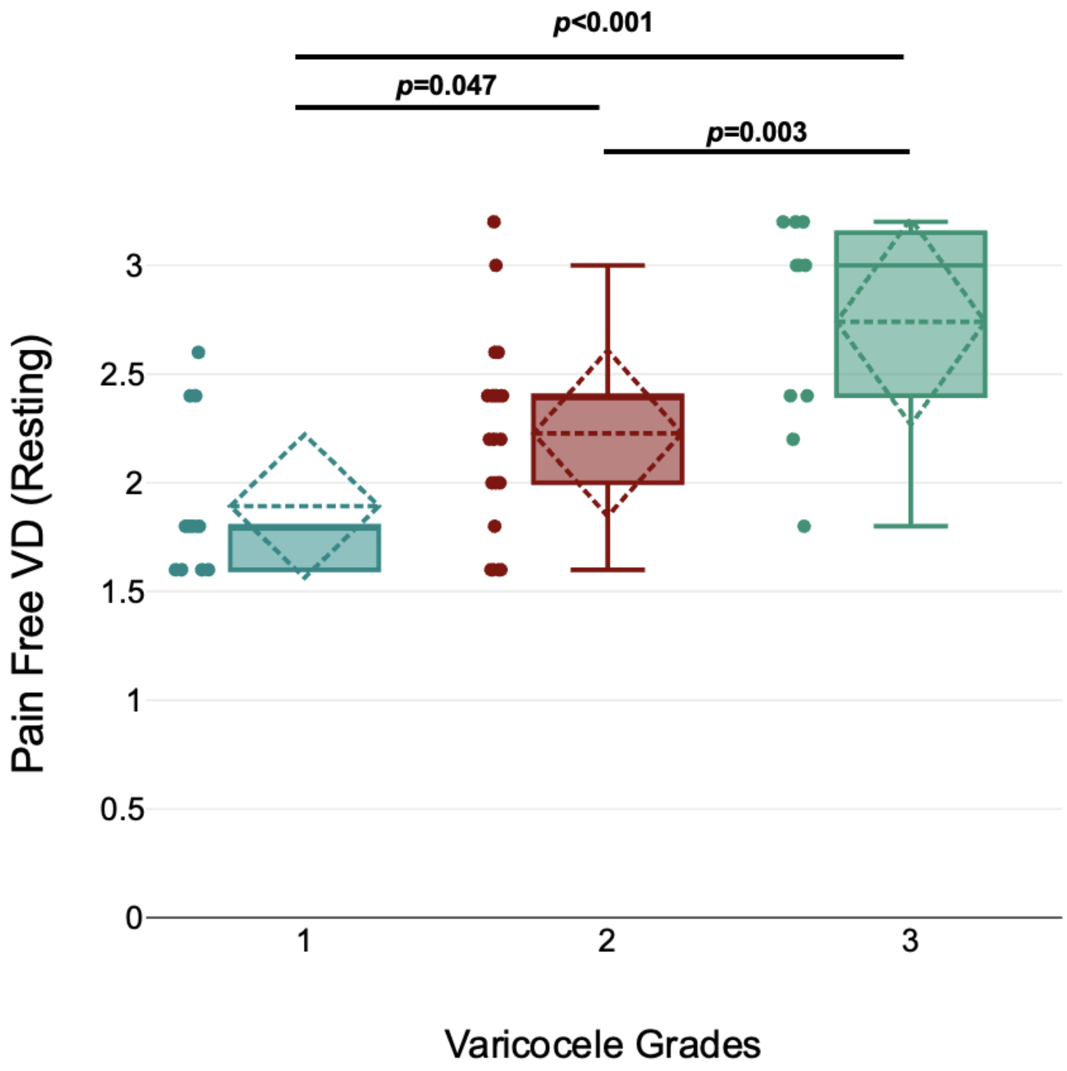

Supplementary Figure S4. Comparison of pain-free state venous diameters (VD) at rest across different varicocele grades. The boxplots depict the median, interquartile range (IQR), and outliers for Grades 1, 2, and 3. Statistical analysis indicates significant differences in venous diameters between Grades 1 and 2 (p=0.047), Grades 2 and 3 (p=0.003), and Grades 1 and 3 (p<0.001).

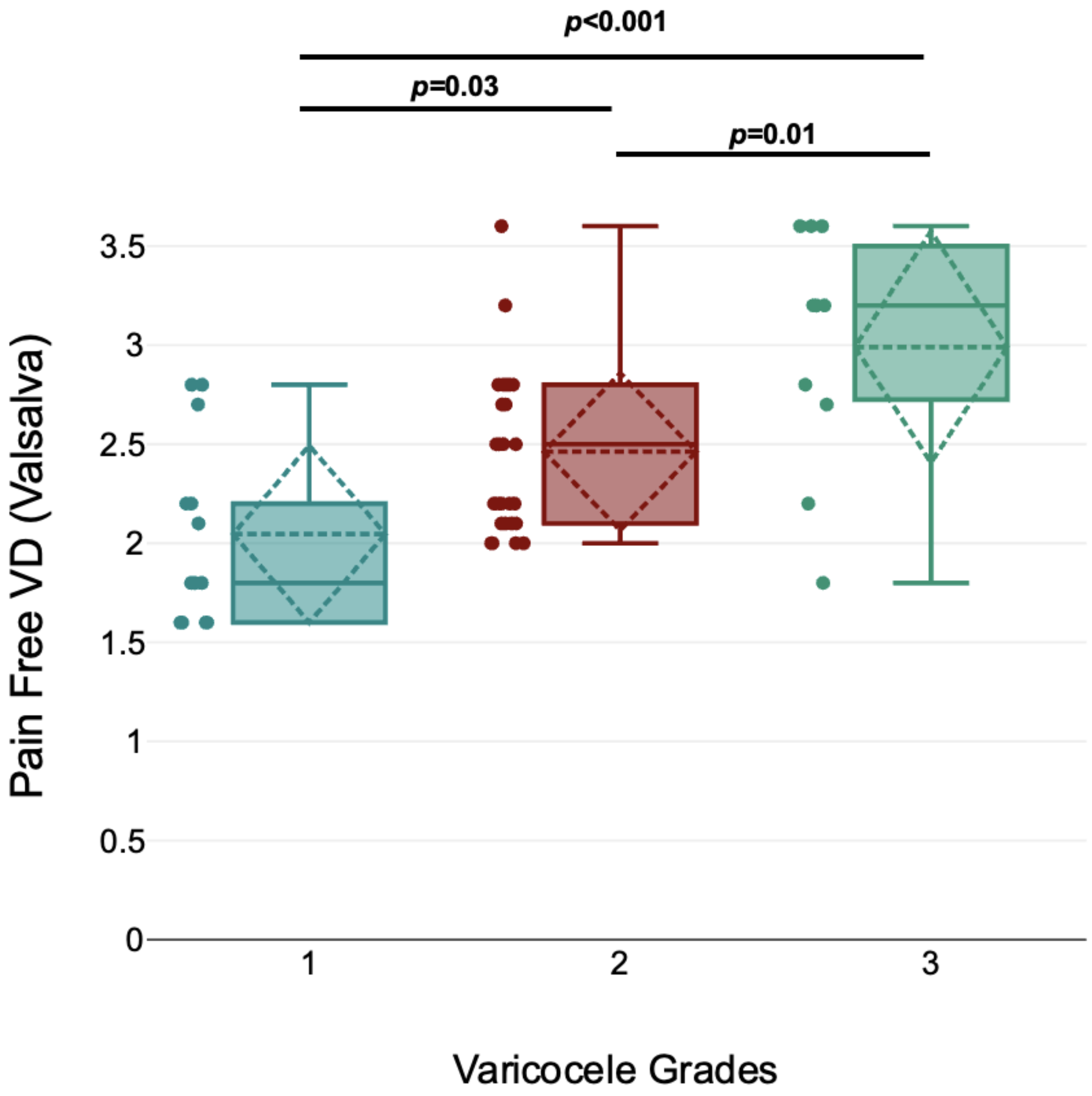

Supplementary Figure S5. Comparison of pain-free venous diameters (VD) during the Valsalva maneuver across different varicocele grades. The boxplots illustrate the median, interquartile range (IQR), and outliers for Grades 1, 2, and 3. Statistical analysis reveals significant differences in venous diameters between Grades 1 and 2 (p=0.03), Grades 2 and 3 (p=0.01), and Grades 1 and 3 (p<0.001).

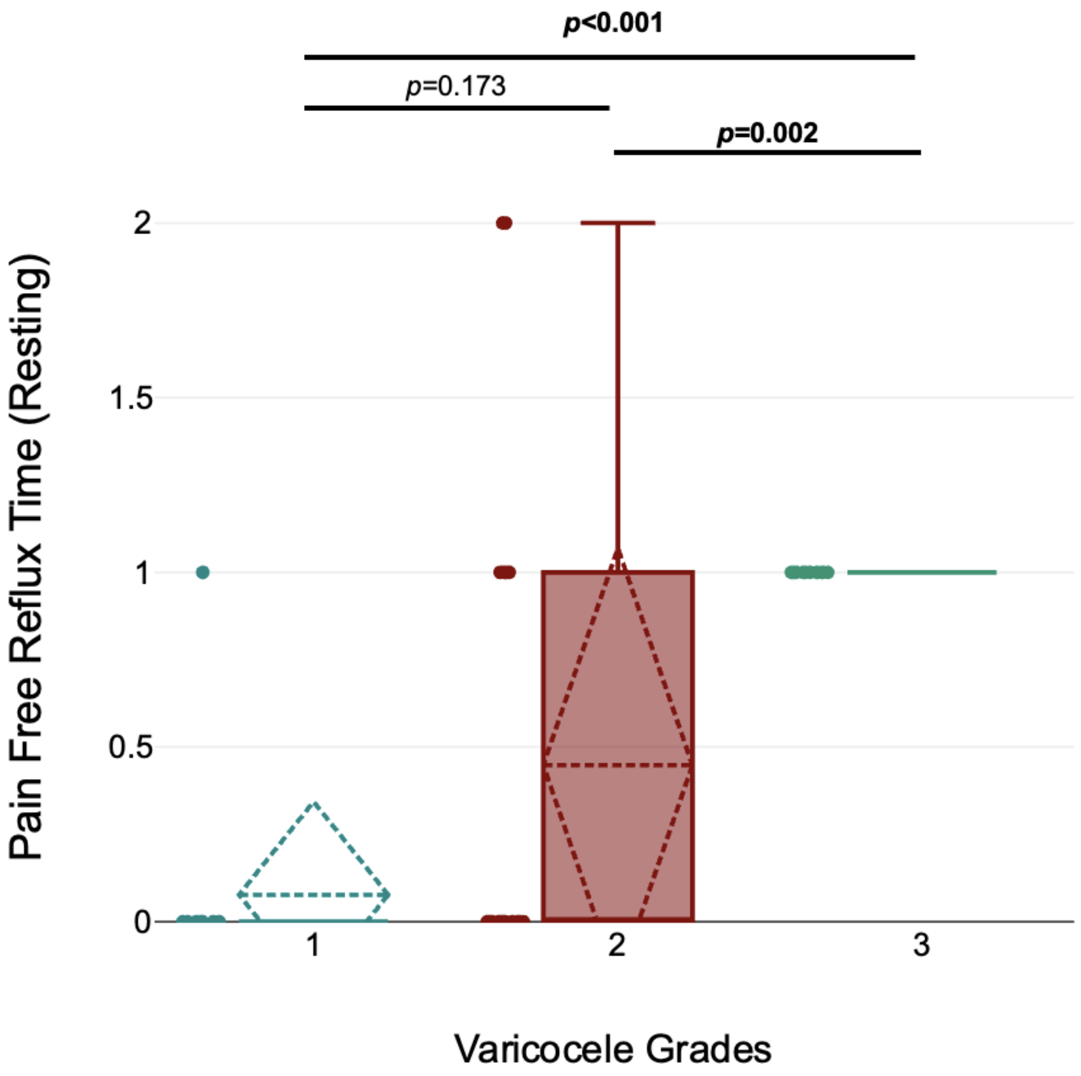

Supplementary Figure S6. Comparison of pain-free reflux time (RT) at rest across different varicocele grades. The boxplots display the median, interquartile range (IQR), and outliers for Grades 1, 2, and 3. Statistical analysis shows significant differences in reflux time between Grades 1 and 3 ($p<0.001$) and Grades 2 and 3 ($p=0.002$), while the difference between Grades 1 and 2 is not significant ($p=0.173$).
